# A Wideband Eight-Port MIMO Antenna with Reduced Mutual Coupling for Future 5G mm-Wave Applications

**DOI:** 10.3390/s25020484

**Published:** 2025-01-16

**Authors:** Muhammad Kabir Khan, Shaobin Liu, Muhammad Irshad Khan

**Affiliations:** College of Electronic and Information Engineering, Nanjing University of Aeronautics and Astronautics, Nanjing 210016, China; kabirnawab@nuaa.edu.cn (M.K.K.);

**Keywords:** MIMO, mm-wave, isolation, diversity characteristics, gain

## Abstract

An eight-element MIMO antenna with a neutralization line was utilized for future 5G mm-wave applications. The MIMO configuration was designed for two ports, four ports and eight ports to validate the desired impedance and radiation characteristics. The measured results in terms of MIMO and scattering parameters correlate well with the simulated one. The printed eight-port antenna was a miniaturized size of 44 × 70 × 0.8 mm^3^. Roger RT/duroid 5880 substrate was used to print antennas. The presented antenna produced a vast bandwidth of 18 GHz, varying from 28 to 46 GHz, and achieved a reduced mutual coupling of 30 dB with 6.8–8.5 dBi gain. The eight-port antenna is compared with contemporary antennas considering size, isolation, impedance bandwidth, diversity characteristics and radiation properties, confirming that the presented antenna is a promising candidate for future 5G mm-wave applications.

## 1. Introduction

The growing customer demand for higher-speed mobile internet data poses a challenge for network providers. In today’s digital landscape, where many applications rely on cloud or online services, there is a pressing need for uninterrupted, low-latency data connectivity, a demand that 5G technology aims to fulfill [1,2]. Presently, numerous countries worldwide are in the process of implementing 5G technology to cater to the needs of their customers. The International Telecommunication Union (ITU) estimated that by 2021, approximately 4.9 billion people worldwide, constituting roughly 60% of the global population, would be using the internet. However, as of 31 December 2021, the number of internet users globally reached 5.2 billion. Currently, 4G LTE (Long-Term Evolution) is facing bandwidth saturation levels owing to its limited capacity and growing number of users. The importance of advancing antenna technology for 5G’s mm-wave technology has become extremely critical in the recent past. To address the challenges posed by atmospheric and propagation losses, 5G mm-wave systems require the use of wide-band antennas with high gain. There have been numerous study initiatives to improve patch antenna gain and bandwidth designed for mm-wave applications [3,4,5,6,7]. Employing mm-wave frequencies in evolving 5G network systems has immense potential to significantly increase channel capacity compared to existing wireless networks, especially when combined with MIMO technology. Hence, the interest in mm-wave MIMO antennas is raising user demands for high throughput, requiring close spacing of antenna elements for miniaturization. Nonetheless, designing mm-wave antennas with MIMO applications poses a challenge for antenna engineers due to the unneeded mutual coupling between antenna’s components. To tackle this challenge, various techniques in recent years have been extensively described to reduce mutual coupling between ports [8,9,10,11,12].

Recently, a plethora of MIMO antenna designs have been provided in the literature to achieve different frequency bands, including WiMAX, WiFi, WLAN, Ku band, K band, X band, UWB and mm-wave applications [13,14,15,16,17,18,19,20,21,22,23,24,25,26,27,28,29,30,31,32,33,34,35]. In article [18], an eight-port antenna with UWB application is introduced. Two C- and T-shaped slots in a 5G metal frame are used to optimize the bandwidth from 3.3–6 GHz with 18 dB enhanced isolation. The 150 × 75 × 7 mm^3^-size antenna achieves a total efficiency of 40–90% with envelope correlation coefficient (ECC) ≤ 0.05. A four-port PIFA MIMO antenna with vast bandwidth is presented to cover 5G NR bands in 5G smartphones [19]. The antenna pair is 30 × 7 mm^2^ in size, achieving a 3.3–7.5 GHz bandwidth. The PIFA MIMO antenna achieves > 10 dB isolation with ECC ≤ 0.05. A four-element, eight-port diversity antenna is presented for 5G handheld cellular IoT applications [20]. The dimension of the proposed antenna is taken as a modern mobile set. The radiating elements are placed at the corners of two feeding ports each. The antenna works at a bandwidth of from 2.4 to 3.8 GHz, with isolation ≤ 13 dB. The secured ECC is <0.03, with 3.2–5 dBi peak gain. A compact 3D eight-port antenna array is examined in [21]. The array consists of eight radiating elements, in which four radiating elements are configured in planar configuration, and the other four are placed perpendicular to it. The antenna obtains an enhanced isolation of 15 dB from 3 to 11 GHz bandwidth. The designed 50 × 50 × 25 mm^3^ dimension antenna is printed on Roger TMM4 laminate. An eight-element, fifth-generation antenna is designed for 5G smartphone applications in [22]. The 150 × 75 × 7.8 mm^3^-size proposed antenna achieves 3.2–6 GHz frequency with an isolation of ≤12.6 dB, an efficiency of 38–83% and ECC ≤ 0.31. An eight-component MIMO antenna setup is recommended to ensure future handsets in article [23]. The |S_11_| ≤ 10 dB in 3.4–3.6 GHz band has an isolation > 17.5 dB between ports. The 150 × 80 × 0.5 mm^3^-dimension antenna achieves an efficiency of >62% and an ECC of <0.05. In article [24], an eight-port antenna with CP functionality is presented to operate in 5–6 GHz bandwidth with an isolation of 8 dB. The designed antenna has a 4.9 dBi peak gain and ECC < 0.1. A compact dual-port antenna with wide bandwidth is presented for 38 GHz upcoming 5G applications in [25]. Roger RT/duroid 5880 substrate is used to print 35 × 30 × 0.8 mm^3^ dimension antenna. The antenna achieves an 8.9 GHz operating frequency from 34.8 to 43.7 GHz. The ECC < 0.0002, diversity gain (DG) ≥ 9.999 dB with 10.24 dBi peak gain and isolation ≤ 20 dB. A CPW-fed four-element flexible antenna is printed for future wearable 5G/Wi-Fi 6E applications in [26]. In study [27], a 28/38 GHz crescent-shaped antenna is printed to achieve 5G applications. The 60 × 60 × 0.508 mm^3^-size antenna is orthogonally placed to obtain an operating bandwidth of 3.05 GHz and 2.41 GHz with good diversity performance. In article [28], a MIMO Vivaldi antenna is fabricated for upcoming mm-waveband applications. In this study, the peak gain is improved by using a bi-axial antistrophic metasurface. A MIMO resonator antenna with 5G applications and improved isolation is presented [29]. The antenna works from 27.25 to 28.59 GHz, located by the FCC for 5G communications. In study [30], a new nature-inspired antenna with a novel MIMO structure is fabricated for 5G communications. An isolation of 17 dB is achieved from 26 to 30 GHz working bandwidth. The antenna also obtains 7.8 dBi gain and 95% efficiency. In [31], a semi-flexible mm-wave dual-port antenna is proposed for wearable applications. The size of the presented antenna is 22.5 × 36 mm^2^ with a 24–31 GHz secured band. The proposed antenna secures an enhanced isolation of 20 dB. A 4 × 4 low-profile circularly polarized, slot-coupled antenna array is presented for emerging mm-wave applications in [32]. The antenna achieves an operating bandwidth of 28–30 GHz. To secure a 26.5–29.5 GHz operating bandwidth, a 1 × 8 multimode antenna array is presented for mm-wave terminals in [33]. A helical-inspired MIMO end-fire antenna is presented for 5G diversity applications in [34]. The 15 × 25 mm^2^ antenna is printed on Roger RO4003. The antenna works from 26.25 to 30.14 GHz. The antenna has ECC < 0.005 with DG > 9.95 dB. A novel wideband MIMO patch antenna is fabricated to achieve 28 GHz 5G application in [35]. To obtain 84% radiation efficiency, 26.5–32.9 GHz-wide bandwidth and 5 dBi gain, rectangular slots are defected in the radiator and ground of the antenna. The dual-port antenna has dimensions of 30 × 15 × 0.25 mm^3^ and is printed on ROGER 4003.

In the literature discussed above, most of the researchers presented two-port and four-port MIMO antennas with lower frequency ranges, non-connected ground planes, low gain and efficiency, poor diversity performance, large size, complex circuitry and narrow operating bandwidth. To address the above challenges, an 8 × 8 MIMO antenna is presented for mm-wave applications. The presented antenna consists of a four-unit cell with connected defected ground structures (DGS). Every unit cell consists of two radiating elements containing a stub inside and a neutralization line block. The radiating elements and neutralization line block is positioned on the front side and DGS on the backside. The antenna secures a wide bandwidth of 28–46, covering 5G mm-wave communications [36,37]. The neutralization line block and four half-circle ground slots are incorporated for isolation enhancement. The proposed MIMO antenna has secure good radiation performance, with a radiation efficiency of 91–97% and a peak gain of 6.8–8.5 dBi. The mm-wave MIMO antenna has a good diversity performance of ECC < 0.0001 and DG > 9.999 dB. To validate and examine the antenna further, the 44 × 70 × 0.8 mm^3^ miniaturized antenna is printed to confirm the simulated outcomes.

## 2. Antenna Design

An eight-port MIMO antenna with improved bandwidth was designed for future 5G communication. The printed antenna features a unique design printed on Roger RT/duroid 5880 substrate in Shenzhen Bomin Electronic Technology Co., Ltd., Shenzhen, China. The design antenna operates from 28 to 46 GHz with 30 dB enhanced isolation. This section is divided into four parts, discussing single-, dual-, quad- and eight-port MIMO antennas.

### 2.1. Single-Port Rectangular-Shaped MIMO Antenna

Equation (1) is used to achieve the required geometry parameters of a rectangular-shaped antenna [38].

(1) $$W=\frac{c}{2f\sqrt{\frac{\left(\varepsilon_{r}+1\right)}{2}}}$$

**Figure 1:**
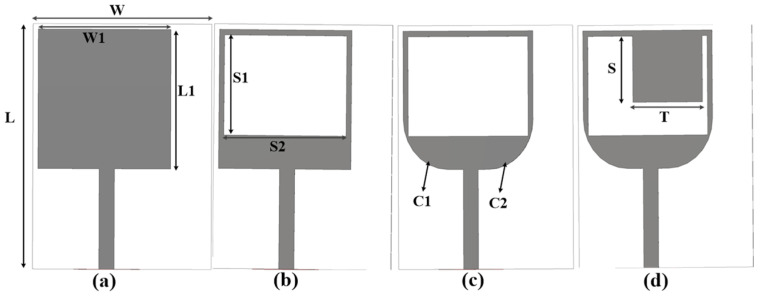
Design development of the single-port antenna. (**a**) Rectangular antenna, (**b**) rectangular slot antenna, (**c**) rectangular chamfered edge antenna, and (**d**) proposed single-port antenna.

Here, $\varepsilon_{r}\;$ is the dielectric constant, c is the speed of light and f  is the resonant frequency. The design development of the antenna is given in Figure 1. The size of the single-port antenna is 17.5 × 22 × 0.8 mm^3^.

**Figure 2:**
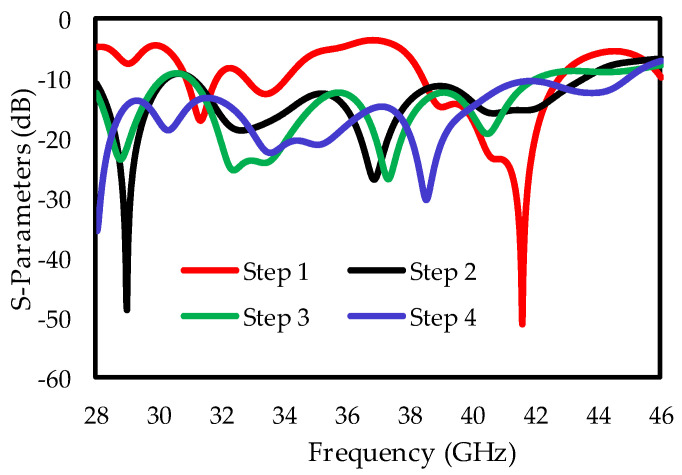
S-parameter results for the development stages of the single-port antenna.

In the first step, a simple rectangular antenna of size 17.5 × 22 × 0.8 mm^3^ is simulated, and the results are given in Figure 2. To achieve the desired S-parameter, a square-shaped slot with size S1 = 9 mm and S2 = 12 mm was etched in the radiator to enhance the bandwidth. The lower edges (C1 and C2) of the radiator were chamfered by 4.5 mm, and a rectangular-shaped stub of size S = 7 mm and T = 6 mm was added to secure a 28–46 GHz bandwidth. The |S_11_| results for the design development stages are presented in Figure 2, and it is clear that the antenna used in step (d) achieves a vast bandwidth of 18 GHz, varying from 28 to 46 GHz for the proposed single-element design.

### 2.2. Dual-Port MIMO Antenna

**Figure 3:**
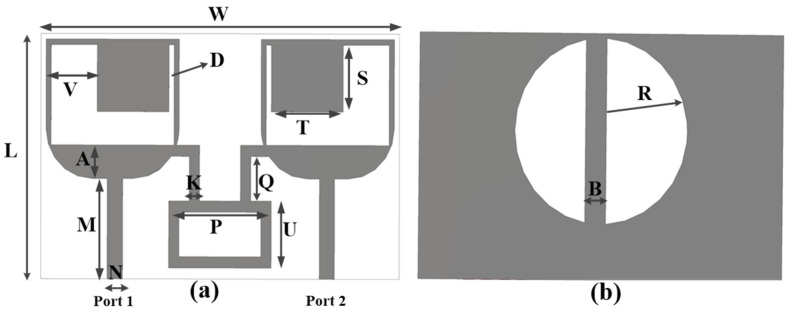
(**a**) Front and (**b**) back sides of the dual-component MIMO antenna.

The dual-port novel-shaped antenna was composed of two similar novel-shaped radiating elements with a U-shaped slot and chamfered edges. The dual-port antenna had a common ground, with two vertical half-circle slots, and a neutralization block was added to enhance operating bandwidth and isolation. The front and back sides of the proposed antenna are shown in Figure 3.

The two elements, slotted rectangular-shaped radiating components of dimension 35 × 22.5 × 0.8 mm^3^, were placed contiguously for better component spacing.

**Figure 4:**
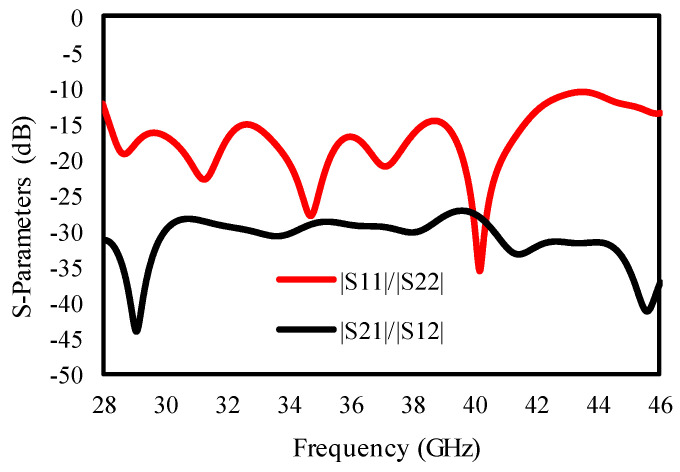
The dual-port MIMO antenna S-parameter’s results.

A slot with width V and D was etched, a square stub with width T × S was added, and the lower edges of the rectangular-shaped radiating elements were chamfered by 4.5 mm each to further increase the bandwidth and isolation. A 50 Ω microwave strip line (M = 9 × N = 1.48) was used for antenna–feeding. The radius of a half circle is given by (R). The antenna resonates from 28 to 46 GHz, as is clear from Figure 4. To achieve an enhanced isolation, a metallic strip (neutralization line) was added between the radiating element. The length of the neutralization line is given by (U) and the width by P. With the use of a neutralization line, 30 dB enhanced isolation was secured without any other complex circuits, as is clear from Figure 4.

**Figure 5:**
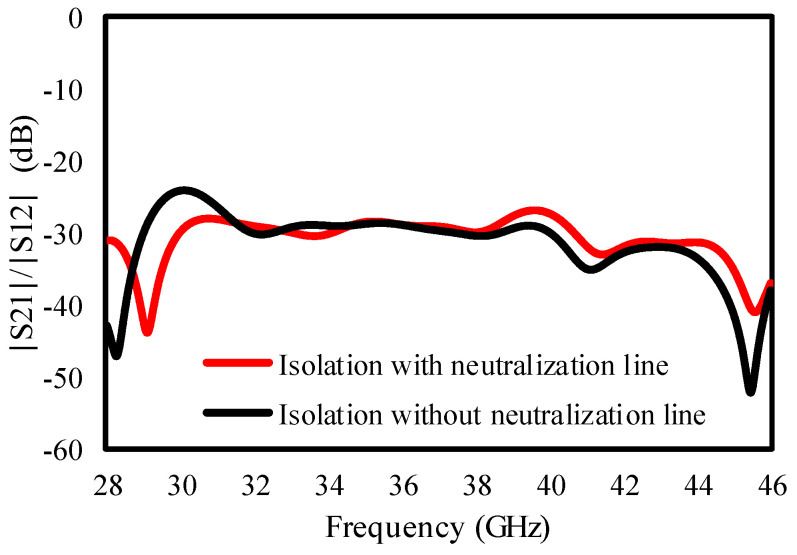
Isolation result of dual-port antenna with and without neutralization line.

The effect of the neutralization line on the isolation between ports is given in Figure 5, and Figure 5 confirms that the isolation in the lower frequencies is increased from 25 dB to 30 dB.

**Figure 6:**
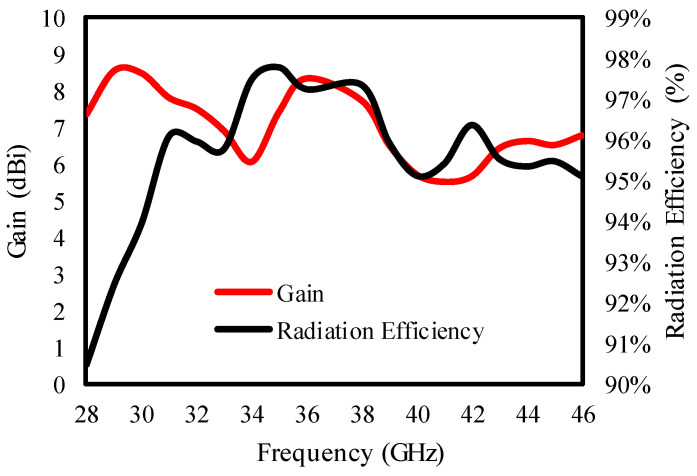
Radiation efficiency and gain of the dual-port MIMO antenna.

The dual-port antenna secures a peak gain of 6.8 to 8.5 dBi with a radiation efficiency of 91 to 97%, as is clear from Figure 6.

**Figure 7:**
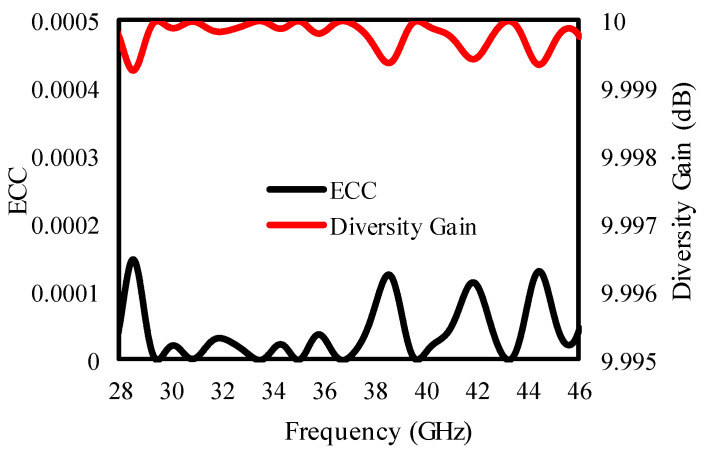
ECC and DG of the suggested antenna.

The presented antenna demonstrates a good diversity performance of ECC < 0.0001 and DG < 9.999 dB, as is clear from Figure 7.

**Figure 8:**
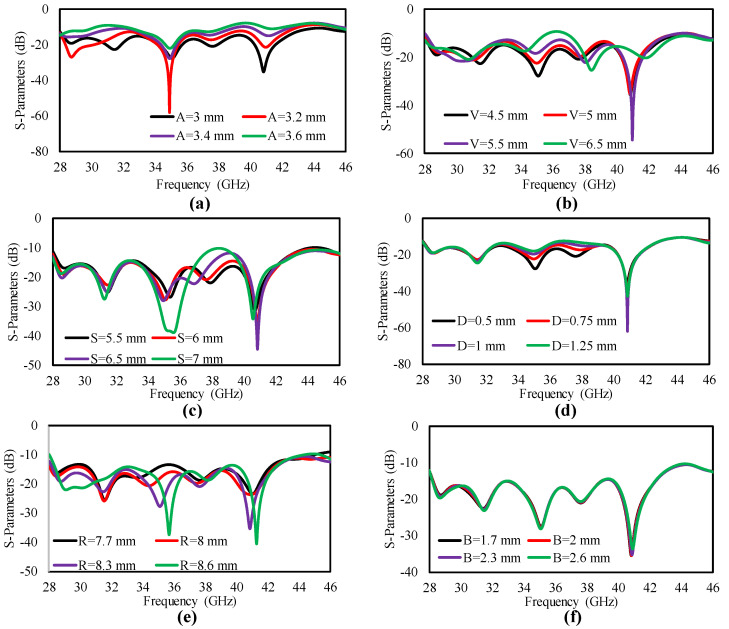
Reflection coefficient of the MIMO antenna. (**a**) Rectangular A, (**b**) slot V (**c**) slot D, (**d**) stub S, (**e**) ground slot R and (**f**) ground-stub B.

The optimization and parametric analysis were carried out in CST Studio for optimum values for the antenna parameters. The dimensions of the lower rectangle (A), the slot (V), the thin slot (D), the length of the stub (S), the radius of the half circle (R) and the ground stub (B) were altered to analyze their effect on the antenna performance, as shown in Figure 8.

The dimension of the rectangular A was varied from 3 to 3.6 mm in steps of 0.2 mm, and all the other parameters were kept constant. It is clear from Figure 8a that the antenna achieved an improved bandwidth with a 3 mm size. The effect of slot V is studied in Figure 8b. The width of slot V was varied from 4.5 to 6.5 mm in steps of 0.5 mm, and Figure 8b shows that the designed antenna obtained an improved bandwidth with size 4.5 mm. The effect of stub (S) on antenna reflection coefficient is given in Figure 8c, and it is clear that the antenna demonstrated a good performance with a 7 mm size. The width of the thin slot (D) was varied from 0.5 to 1.25 mm in steps of 0.25 mm, with all other antenna parameters kept constant. Figure 8d clearly shows that the antenna achieved a good performance with size 0.5 mm. The radius (R) of the ground half-circle slot is given in Figure 8e. The size of the radius (R) was varied from 7.7 to 8.6 mm in steps of 0.3 mm, and it is clear from Figure 8e that the antenna achieved a good performance with size 8.6 mm. The ground stub size effect was nominal, as is clear from Figure 8f.

After analyzing the optimal values for antenna parameters, the suggested antenna’s complete dimensions are given in Table 1.

### 2.3. Quad-Port Ring-Shaped MIMO Antenna

Shannon’s theorem states that channel capacity experiences a notable increase with an increase in components [39].

**Figure 9:**
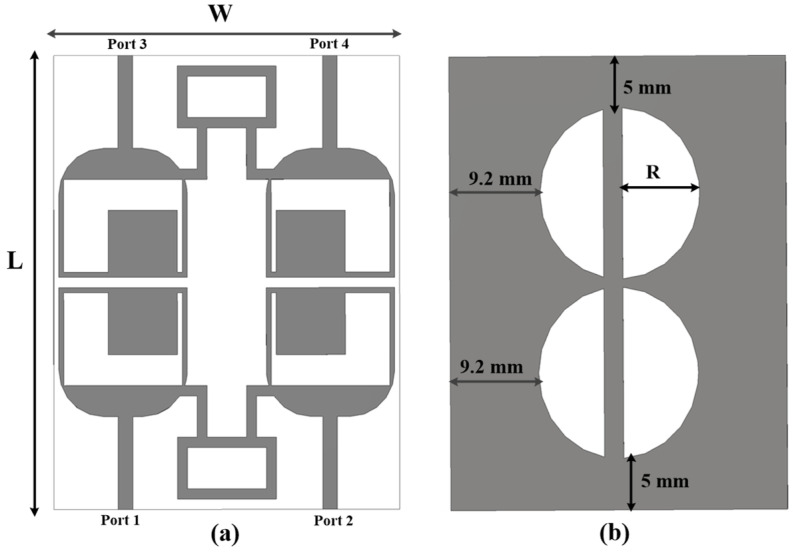
Quad-port MIMO antenna design. (**a**) Top and (**b**) bottom view.

Based on this study, the dual-port antenna was further modified to a quad-element antenna given in Figure 9. All the parameters of the radiating elements were the same as the antenna in Figure 3. The four-element antenna’s dimensions were 35 × 44 × 0.8 mm^3^, with four half-circle slots in the antenna’s ground for bandwidth and isolation enhancement. The four-port antenna was divided into two blocks of dual-port antennas with a neutralization line for a better isolation enhancement of 30 dB.

**Figure 10:**
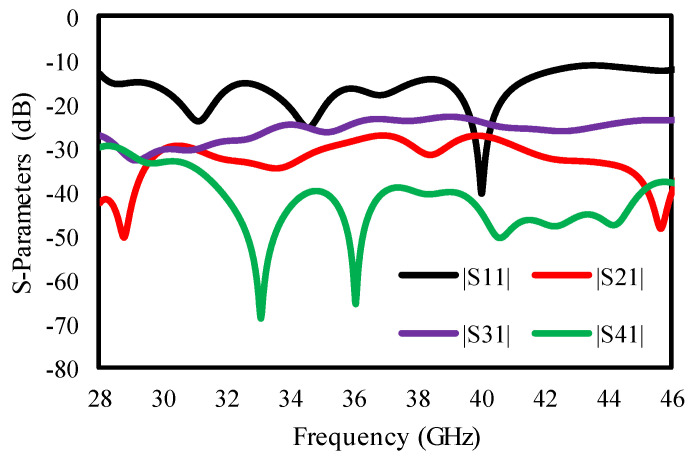
S-parameter results for quad-port MIMO antenna.

The reflection coefficient of the quad-port antenna was <−10 dB in the 28 to 46 GHz frequency range, with an isolation enhancement of <30 dB. Figure 10 reveals the S-parameter results of the quad-element MIMO antenna.

**Figure 11:**
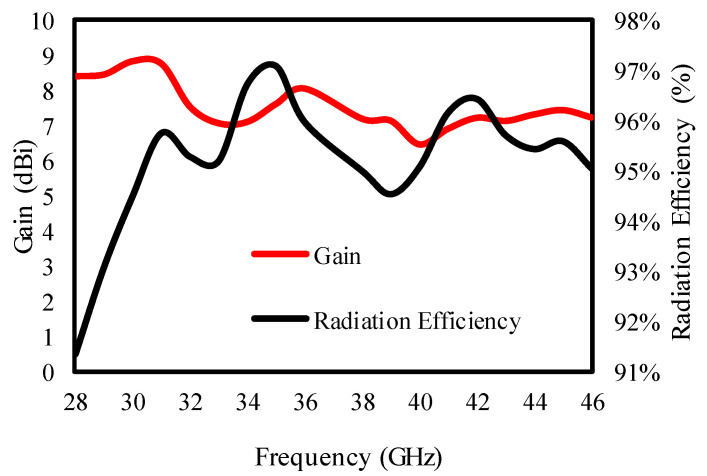
Peak gain and radiation efficiency plot.

Figure 10 demonstrates that the quad-element MIMO antenna achieves an 18 GHz vast bandwidth, varying from 28 to 46 GHz, with an enhanced isolation of 30 dB. Figure 11 plots gain and radiation efficiency to further confirm the antenna’s capabilities, securing a gain from 6.8 to 8.5 dBi with 91–97% radiation efficiency.

**Figure 12:**
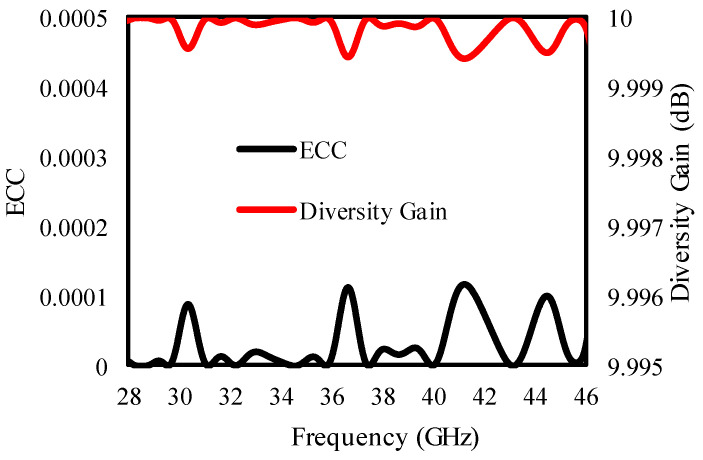
ECC and DG of the quad-port antenna.

Figure 12 presents the diversity performance of the quad-element antenna for further examination of its accuracy. The diversity performance of the MIMO antenna is calculated using ECC and diversity gain [40]. It is necessary for MIMO antennas to have ECC values below 0.5, and this is evaluated in Equation (2).

(2) $$ECC=\frac{\left|\iint_{4\pi }\left[{\vec{F}}_{1}(\theta ,\varphi \right)•{\vec{F}}_{2}\left(\theta ,\varphi \right)\right]d\Omega {|}^{2}}{\iint_{4\pi }|{\vec{F}}_{1}(\theta ,\varphi ){|}^{2}d\Omega \iint_{4\pi }|{\vec{F}}_{2}(\theta ,\varphi ){|}^{2}d\Omega }$$

${\vec{F}}_{1}(\theta ,\varphi )$ is the field radiation pattern of port 1, ${\vec{F}}_{2}(\theta ,\varphi )$ is the field radiation pattern of port 2, and • is the Hermitian product.

(3) $$DG=10\sqrt{1-(ECC{)}^{2}}$$

The quad-element MIMO antenna’s diversity performance, considering ECC and DG, is examined in Figure 12, and Figure 12 confirms that the antenna secures an excellent diversity performance with ECC ≤ 0.0001 and diversity gain ≥ 9.999 dB.

### 2.4. Eight-Element, Slotted Rectangular-Shaped MIMO Antenna Structure

**Figure 13:**
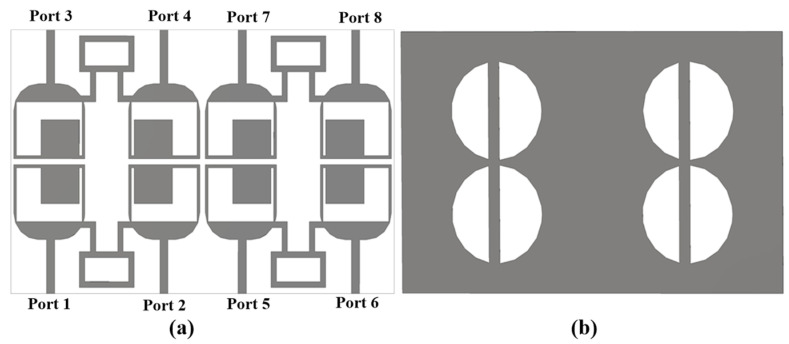
Antenna’s (**a**) front and (**b**) back views.

To obtain a high data rate and channel capacity, the four-element antenna was further modified to an eight-element antenna, with the radiator footprint kept the same as the four-element antenna produced in Figure 9, with a final size of 70 × 44 × 0.8 mm^3^. The proposed eight-element antenna consisted of eight slotted rectangular-shaped radiators with chamfered lower edges and was fed by 50 Ω microstrip lines. The radiator shared a common ground plane with four half-circles slots. Figure 13 shows both front and back views of the suggested eight-element MIMO antenna. The eight-port antenna was divided into four blocks of dual ports with a neutralization line between them for better isolation results.

**Figure 14:**
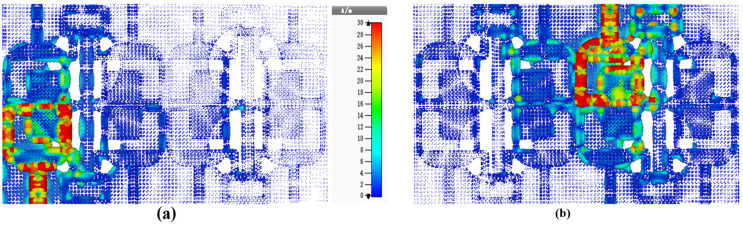
Current distribution of the proposed MIMO antenna at 28 GHz, (**a**) port 1 excited and (**b**) port 7 excited.

Figure 14 illustrates the current distribution of the eight-port antenna for further analysis. When port 1 was excited at 30 GHz, the current distribution revealed that the highest current was concentrated at the edges of radiator 1, strip line 1 and the neutralization block between port 1 and port 2, as shown in Figure 14a, resulting in an enhanced isolation of 30 dB. Therefore, a much-enhanced isolation between ports was achieved. To further analyze the antenna, the current distribution of the antenna is given in Figure 14b while exciting port 7, and it is evident from Figure 14b that the other component was not affected, and enhanced isolation is observed without any extra decoupling structure.

## 3. Results and Discussions

**Figure 15:**
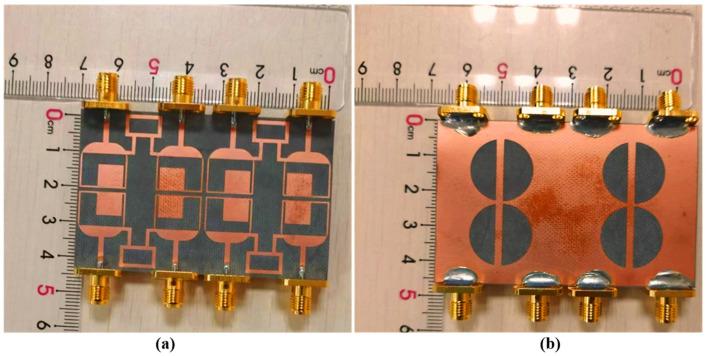
(**a**) Front and (**b**) back view of the fabricated antenna.

The recommended eight-element ring-shaped MIMO antenna is an extension of the dual-port antenna presented in Figure 3. The eight-port antenna was printed and developed to verify the simulated outcomes of the mentioned antenna. It was printed on Roger RT/duroid 5880 substrate. The front and back sides of the printed antenna are given in Figure 15.

### 3.1. Reflection Coefficient

**Figure 16:**
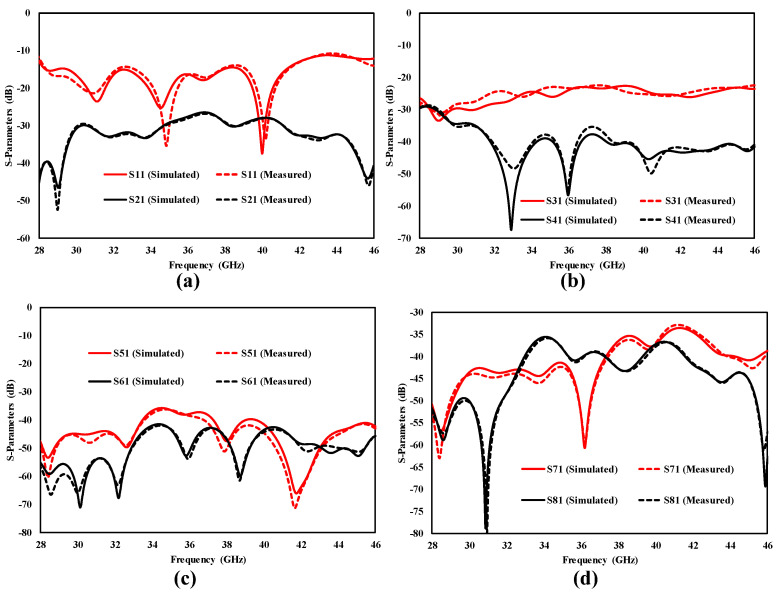
S-Parameters of the proposed MIMO antenna. (**a**) Antenna 1 and antenna 2, (**b**) antenna 1 with antenna 3 and antenna 4, (**c**) antenna 1 with antenna 5 and antenna 6 and (**d**) antenna 1 with antenna 7 and antenna 8.

The eight-port MIMO antenna was printed and developed to examine the simulated results with the measured results. The results show that the antenna achieved a vast bandwidth of 18 GHz, varying from 28 to 46 GHz, with enhanced isolation of 30 dB, as confirmed in Figure 16. Figure 16 demonstrates that both results are nearly the same, with tiny differences due to small defects in the production process and losses of the SMA connecters. When port 1 is excited, the remaining seven ports are individually terminated with a 50 Ω load. Figure 16a illustrates |S_11_| and |S_21_| results of the designed eight-port MIMO antenna. The |S_11_| < −10 dB from 28 GHz to 46 GHz, and |S_21_| < 30 dB, as is clear from Figure 16a. Figure 16b depicts the effect of antenna elements 1 on elements 3 and 4 after stimulating port 1. The MIMO antenna had a measured and simulated isolation level of <40 dB in the operational bandwidth, with the exception of |S_31_| at less than 23 dB. The |S_41_| is ˂ 40 dB over the entire working range. To check the effect of antenna 1 on antennas 5 and 6, the |S_51_| and |S_61_| responses are studied in Figure 16c, and Figure 16c confirms that |S_51_| < 40 dB and |S_61_| < 45 dB. To examine the MIMO antenna further, the effect of antenna 1 on antenna 7 and antenna 8 is given in Figure 16d, and it shows that the proposed MIMO antenna’s |S_71_| < 35 dB and |S_81_| < 36 dB. All antenna elements are identical, giving the same return loss response across all ports.

### 3.2. Radiation Patterns, Peak Gain and Radiation Efficiency

To assess the performance of the antennae further, the radiation patterns, gain and radiation efficiency are measured and discussed in this section.

**Figure 17:**
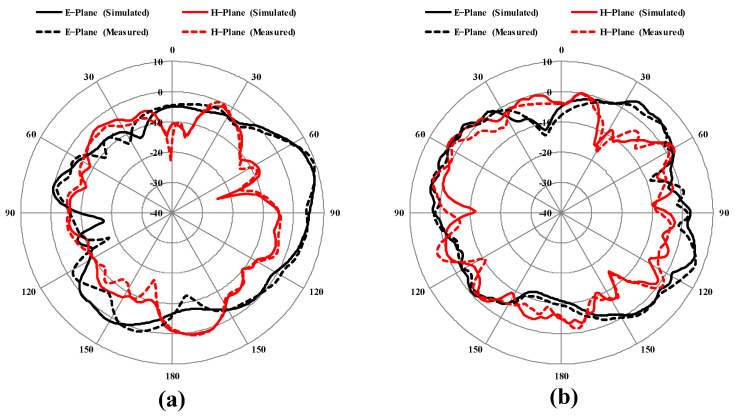
Radiation patterns of presented MIMO antenna at (**a**) 30 GHz and (**b**) 40 GHz.

The radiation patterns in E-plane and H-plane at 30 GHz and 40 GHz are studied in Figure 17. Figure 17 confirms that the intensity of the radiation at 30 GHz in the both E-plane and H-plane is found between 150° and 180°. The intensity of radiation at 40 GHz in the E-plane is analyzed at 60° and 120°, with the radiation intensity in the H-plane at 40 GHz occurring at 150° for exciting port 1 and terminating the remaining ports. For identical antennas, its results are the same but with 90° rotation. The simulated and measured results of the proposed antenna matched well; however, marginal mismatch occurred due to fabrication tolerance, connector loss and noise from the environment during testing.

**Figure 18:**
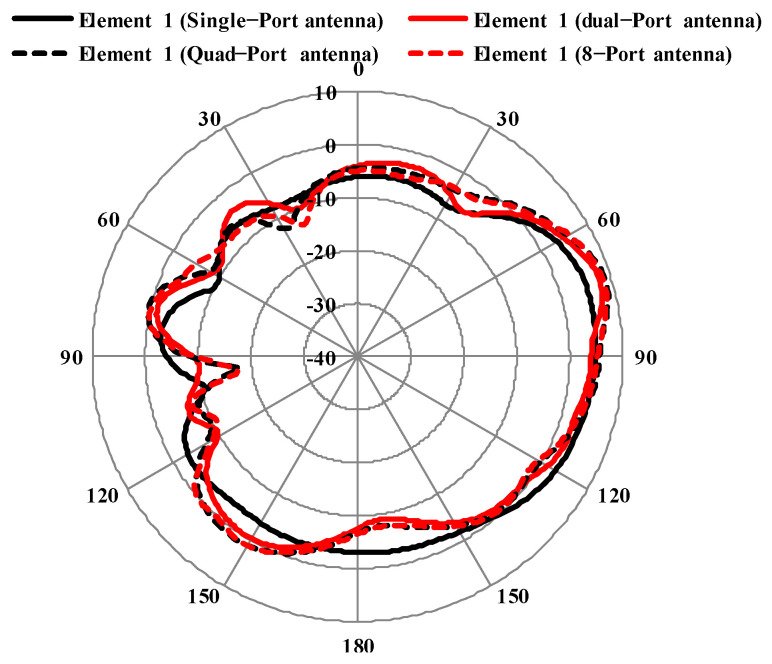
Radiation patterns of element 1 in proposed antenna.

The variation in the radiation patterns of element 1 of single-, dual-, quad- and octa-port antennas is given in Figure 18.

**Figure 19:**
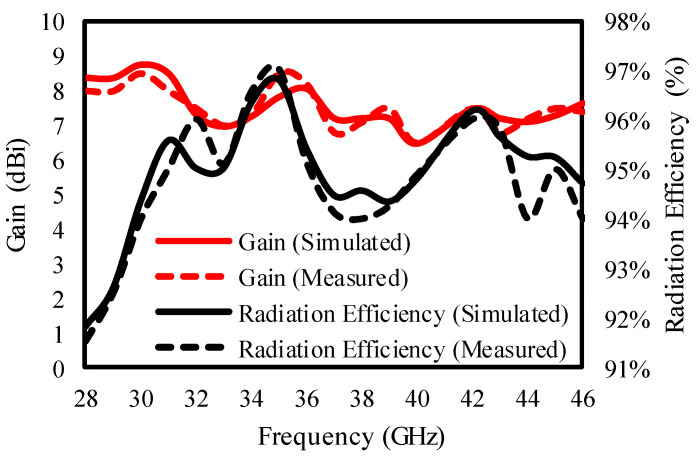
Gain and radiation efficiency of the presented antenna.

The antenna secured 6.8 to 8.5 dBi gain with 91 to 97% radiation efficiency in the entire operating range, as confirmed by Figure 19.

### 3.3. Diversity Performance Analysis

**Figure 20:**
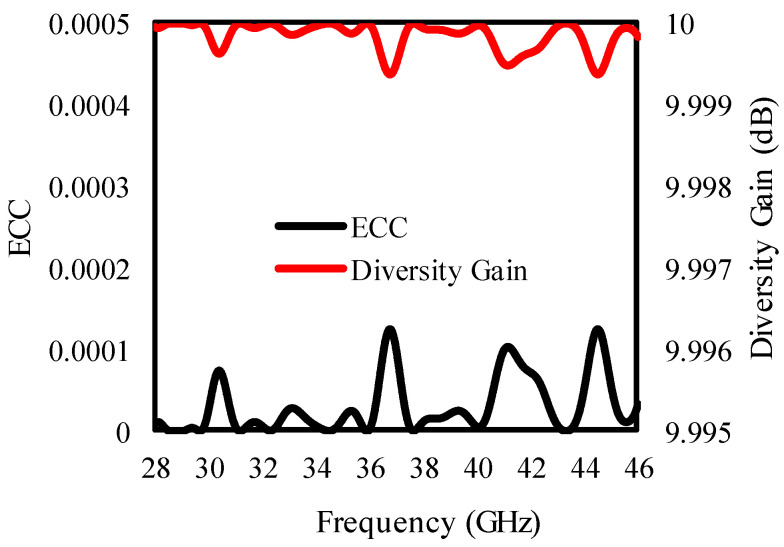
ECC and DG of the presented antenna.

To verify the performance of the designed antenna further, the ECC and DG of the proposed antenna are given in Figure 20. The ECC of the proposed antenna is <0.0001, while the DG of the proposed antenna ≥ 9.999 dB, as is clear from Figure 20.

The performance comparison is demonstrated in Table 2.

## 4. Conclusions

In this research article, the effectiveness of an improved compact mm-wave eight-port MIMO antenna in terms of gain, bandwidth, ECC and isolation was comprehensively investigated. The proposed eight-port ring-shaped antenna has compact dimensions of 70 × 44 × 0.8 mm^3^, achieving a bandwidth of 18 GHz varying from 28 to 46 GHz. The antenna achieved a gain of 6.8 to 8.5 dBi and radiation efficiency of 91 to 97%. The compact size and good diversity show the effectiveness of the tested antenna. The simple circuitry, compact size, enhanced bandwidth, reduced mutual coupling and excellent diversity support that the proposed antenna is a potential candidate in mm-wave MIMO communication for 5G applications.

## Figures and Tables

**Table 1:** The dual-port antenna’s parameters.

Parameter	Size in mm	Parameters	Size in mm
W	35	A	3
L	22	S	7
T	6	R	8.6
V	4.5	M	9
D	0.5	N	1.48
P	8	U	6
Q	6	K	1

**Table 2:** Performance comparison of the antenna.

Ref #	Size (mm)	Elements	Bandwidth (GHz)	Mutual Coupling (dB)	Gain (dBi)	Efficiency (%)	ECC
[20]	60 × 120	8	1.4	13	3.2–5	…	≤0.03
[21]	50 × 50	8	8	15	…	…	0.5
[22]	150 × 75	8	2.7	>12.6	…	38–83	0.31
[25]	35 × 30	4	8.9	20	10.24	…	<0.0002
[26]	56 × 56	4	5.19	20	1.8–3.2	80	<0.13
[27]	60 × 60	4	3.05 & 2.41	>30	8.14 & 8.04	99.5 & 70	<0.0035
[30]	25 × 15	4	4	17	7.8	95	0.0001
[34]	15 × 25	2	3.89	30	5.83	88	<0.005
[35]	30 × 15	2	6.4	35.8	5.42	83	<0.005
Proposed work	44 × 70	8	18	30	6.8–8.5	91–97	≤0.0001

## Data Availability

Data are contained within the article.

## References

[1] Hong W. (2017). Solving the 5G Mobile Antenna Puzzle: Assessing Future Directions for the 5G Mobile Antenna Paradigm Shift. IEEE Microw. Mag..

[2] Federal Communications Commission (2019). FCC Takes Steps to Make Millimeter Wave Spectrum Available for 5G.

[3] Chen H., Shao Y., Zhang Y., Zhang C., Zhang Z. (2019). A Low-Profile Broadband Circularly Polarized mmWave Antenna with Special-Shaped Ring Slot. IEEE Antennas Wirel. Propag. Lett..

[4] Kim J., Lee H.L. (2022). High Gain Planar Segmented Antenna for mmWave Phased Array Applications. IEEE Trans. Antennas Propag..

[5] Zheng Q., Guo C., Ding J., Vandenbosch G.A.E. (2019). Dual-band metasurface-based CP low-profile patch antenna with parasitic elements. IET Microw. Antennas Propag..

[6] Ozpinar H., Aksimsek S., Tokan N.T. (2020). A Novel Compact, Broadband, High Gain Millimeter-Wave Antenna for 5G Beam Steering Applications. IEEE Trans. Veh. Technol..

[7] Chopra R., Kumar G. (2019). Series-Fed Binomial Microstrip Arrays for Extremely Low Sidelobe Level. IEEE Trans. Antennas Propag..

[8] Karimian R., Kesavan A., Nedil M., Denidni T.A. (2017). Low-Mutual-Coupling 60-GHz MIMO Antenna System with Frequency Selective Surface Wall. IEEE Antennas and Wireless Propag. Lett..

[9] Al-Hasan M., Mabrouk I.B., Almajali E.R.F., Nedil M., Denidni T.A. (2019). Hybrid isolator for mutual-coupling reduction in Millimeter-wave MIMO antenna systems. IEEE Access.

[10] Pan Y., Qin X., Sun Y.-X., Zheng S.Y. (2019). A Simple Decoupling Method for 5G Millimeter-Wave MIMO Dielectric Resonator Antennas. IEEE Trans. Antennas Propag..

[11] Li M., Jamal M.Y., Jiang L., Yeung K.L. (2021). Isolation enhancement for MIMO patch antennas sharing a common thick substrate: Using a dielectric block to control space-wave coupling to cancel surface-wave coupling. IEEE Trans. Antennas Propag..

[12] Gao D., Cao Z.-X., Fu S.D., Quan X., Chen P. (2020). A novel slot-array defected ground structure for decoupling microstrip antenna array. IEEE Trans. Antennas Propag..

[13] Khan M.K., Feng Q. (2022). Design Validation of UWB MIMO Antenna with Enhanced Isolation and Novel Strips for Stop-Band Characteristics. Entropy.

[14] Zhang L., Feng Q., Khan M.K. (2022). Design of a Novel Circularly Polarized MIMO Antenna with Enhanced Isolation for Ultra-Wideband Communication. Appl. Comput. Electromagn. Soc. J. (ACES).

[15] Sohi A.K., Kaur A. (2021). Sextuple band rejection functionality from a compact Koch anti-snowflake fractal UWB-MIMO antenna integrated with split-ring resonators and slots. AEU-Int. J. Electron. Commun..

[16] Modak S., Khan T. (2021). A slotted UWB-MIMO antenna with quadruple band-notch characteristics using mushroom EBG structure. AEU-Int. J. Electron. Commun..

[17] Puskely J., Mikulasek T., Aslan Y., Roederer A., Yarovoy A. (2022). 5G SIW based phased antenna array with cosecant-squared shaped pattern. IEEE Trans. Antennas Propag..

[18] Yuan X.-T., He W., Hong K.-D., Han C.-Z., Chen Z., Yuan T. (2020). Ultra-Wideband MIMO Antenna System with High Element-Isolation for 5G Smartphone Application. IEEE Access.

[19] Yuan X.-T., Chen Z., Gu T., Yuan T. (2021). A Wideband PIFA-Pair-Based MIMO Antenna for 5G Smartphones. IEEE Antennas Wirel. Propag. Lett..

[20] Chattha H.T., Ishfaq M.K., Khawaja B.A., Sharif A., Sheriff N. (2021). Compact Multiport MIMO Antenna System for 5G IoT and Cellular Handheld Applications. IEEE Antennas Wirel. Propag. Lett..

[21] Khan M.S., Rigobello F., Ijaz B., Autizi E., Capobianco A.D., Shubair R., Khan S.A. (2018). Compact 3-D eight elements UWB-MIMO array. Microw. Opt. Technol. Lett..

[22] Jiang J.-Y., Su H.-L. (2022). A Wideband Eight-Element MIMO Antenna Array in 5G NR n77/78/79 and WLAN-5GHz Bands for 5G Smartphone Applications. Int. J. Antennas Propag..

[23] Li Y., Sim C.-Y.-D., Luo Y., Yang G. (2019). High-isolation 3.5 GHz eight-antenna MIMO array using balanced open-slot antenna element for 5G smartphones. IEEE Trans. Antennas Propag..

[24] Biswal S.P., Das S. (2019). Eight-element-based MIMO antenna with CP behaviour for modern wireless communication. IET Microw. Antennas Propag..

[25] Nasri N.E., Ghzaoui M.E., Fattah M. (2024). A quad port MIMO antenna with improved bandwidth and high gain for 38 GHz 5G applications. Frankl. Open.

[26] Peng X., Du C. (2024). A flexible CPW-fed tri-band four-port MIMO antenna for 5G/WIFI 6E wearable applications. AEÜ. Int. J. Electron. Commun..

[27] Cuneray K., Akcam N., Okan T., Arican G.O. (2023). 28/38 GHz dual-band MIMO antenna with wideband and high gain properties for 5G applications. AEU-Int. J. Electron. Commun..

[28] Singh M., Parihar M.S. (2023). Gain Improvement of Vivaldi MIMO Antenna With Pattern Diversity Using Bi-Axial Anisotropic Metasurface for Millimeter-Wave Band Application. IEEE Antennas Wirel. Propag. Lett..

[29] Zhang Y., Deng J.-Y., Li M.-J., Sun D., Guo L.-X. (2019). A MIMO Dielectric Resonator Antenna With Improved Isolation for 5G mm-Wave Applications. IEEE Antennas Wirel. Propag. Lett..

[30] Rahman S., Ren X.C., Altaf A., Irfan M., Abdullah M., Muhammad F., Anjum M.R., Mursal S.N., AlKahtani F.S. (2020). Nature Inspired MIMO Antenna System for Future mmWave Technologies. Micromachines.

[31] Tiwari R.N., Kaim V., Singh P., Khan T., Kanaujia B.K. (2023). Semi-Flexible Diversified Circularly Polarized Millimeter-Wave MIMO Antenna for Wearable Biotechnologies. IEEE Trans. Antennas Propag..

[32] Raeesi A., Palizban A., Ehsandar A., Al-Saedi H., Gigoyan S., Abdel-Wahab W.M., Safavi-Naeini S. (2023). A Low-Profile 2D Passive Phased-Array Antenna-in-Package for Emerging Millimeter-Wave Applications. IEEE Trans. Antennas Propag..

[33] Ansems R., Federico G., Smolders A.B., Caratelli D. (2024). Multimode Phased Antenna Array for mm-Wave User Terminals With Ultrawide-Angle Scanning Capabilities. IEEE Trans. Antennas Propag..

[34] Zahra H., Awan W.A., Ali W.A.E., Hussain N., Abbas S.M., Mukhopadhyay S. (2021). A 28 GHz Broadband Helical Inspired End-Fire Antenna and Its MIMO Configuration for 5G Pattern Diversity Applications. Electronics.

[35] Hussain N., Awan W.A., Ali W., Naqvi S.I., Zaidi A., Le T.T. (2021). Compact wideband patch antenna and its MIMO configuration for 28 GHz applications. AEU-Int. J. Electron. Commun..

[36] Wang L., Liao Q. (2021). Wideband Multibeam SIW Horn Array with High Beam Isolation and Full Azimuth Coverage. IEEE Trans. Antennas Propag..

[37] Chen C., Chen J., Hong W. (2022). Differentially Fed Dual-Polarized 2-D Multibeam Dielectric Resonator Antenna Array Based on Printed Ridge Gap Waveguide. IEEE Trans. Antennas Propag..

[38] Balanis C.A. (2005). Antenna Theory: Analysis and Design.

[39] Jensen M.A., Wallace J.W. (2004). A review of antennas and propagation for MIMO wireless communications. IEEE Trans. Antennas Propag..

[40] Khan M.I., Liu S., Khan M.K., Rahman S.U. (2023). Eight elements mm-wave MIMO antenna for anti-collision radar sensing application with novel hybrid techniques. AEÜ Int. J. Electron. Commun..

